# Genetic Relationships and Molecular Signatures of Divergence in Traditional Landraces and Morphotypes of *Brassica oleracea*

**DOI:** 10.3390/plants14010020

**Published:** 2024-12-25

**Authors:** Diana L. Zuluaga, Nunzio D’Agostino, Emanuela Blanco, Pasquale L. Curci, Gabriella Sonnante

**Affiliations:** 1 Institute of Biosciences and Bioresources, National Research Council, Via Amendola 165/A, 70126 Bari, Italy; diana.zuluaga@ibbr.cnr.it (D.L.Z.); emanuela.blanco@ibbr.cnr.it (E.B.); pasqualeluca.curci@ibbr.cnr.it (P.L.C.); 2 Department of Agricultural Sciences, University of Naples Federico II, Piazza Carlo di Borbone 1, 80055 Portici, Italy; nunzio.dagostino@unina.it

**Keywords:** *Brassica oleracea*, SNPs, local varieties, F*_ST_*, signature of molecular divergence

## Abstract

*Brassica oleracea* is a complex species incorporating a great variety of vegetable types, including cabbage, cauliflower, broccoli, kale, and others. Southern Italy, and especially the Puglia region, is rich in *B. oleracea* landraces. In this study, genotyping-by-sequencing (GBS) was applied to a germplasm panel of 82 samples, mostly landraces and some commercial varieties, belonging to various morphotypes of *B. oleracea*. Population structure (K = 2), principal component (PCA), and phylogenetic analyses resulted in a general subdivision of our samples into two large lineages: the types used for their leaves (LHL) and those consumed for their flower heads (AIL). Going deeper inside, the different morphotypes were mostly grouped into specific clusters, and a clear separation of particular landraces, such as the Mugnoli and Cima nera broccoli, was observed in the structure analysis (K = 7), as well as in the PCA and in the Neighbor-Joining tree. The calculation of the pairwise fixation index (F*_ST_*, threshold > 0.50) between LHL and AIL types (based on population structure analysis at K = 2) provided 456 outlier single nucleotide polymorphisms (SNPs). Among the corresponding orthologs annotated in *Arabidopsis*, we identified several genes involved in flower/inflorescence development, cellular proliferation, etc. Overall, our investigation provides useful information on the knowledge of early domesticated landraces of *B. oleracea* and allows for the attribution of unknown material to the appropriate taxonomical ranking. The analysis of outlier SNPs has highlighted signatures of molecular divergence between LHL and AIL lineages.

## 1. Introduction

*Brassica oleracea*, a key species of the cruciferous family, includes a remarkable variety of vegetable types, comprising cabbages, cauliflower, broccoli, Brussels sprouts, kales, and kohlrabi. The extensive morphological diversity in leaves, flowers, and stems is highly appreciated by consumers. In addition, *B. oleracea* is renowned for its significant nutraceutical value, due to the production of numerous bioactive compounds, particularly the crucifer-specific glucosinolates. These substances offer a broad spectrum of health benefits thanks to their various biological activities [1,2,3,4]. Beyond taxonomic diversity, *B. oleracea* exhibits a substantial range of morphological and molecular variation within its varieties, reflecting a rich potential for selective cultivation and dietary applications [5,6,7].

*Brassica oleracea* has been widely studied, yielding extensive information at the genetic and genomic levels. Genome sequences have been obtained for various types within the species, including a doubled haploid (DH) kale-like type [8], cabbage (subsp. *capitata*) homozygous/DH lines [9,10,11,12,13], broccoli (var. *italica*) [14,15], and inbred lines of cauliflower (var. *botrytis*) [13,16]. A comprehensive *B. oleracea* pan-genome was recently constructed using 27 accessions representing all morphotypes along with wild relatives. This included 22 de novo assembled, chromosome-level genomes, and five previously reported high-quality genomes [17]. Structural variations (SVs) identified across these genomes enabled the characterization of 704 *B. oleracea* accessions, providing deeper insights into the genetic diversity and evolutionary dynamics of the species.

Genotyping-by-sequencing (GBS) provides an excellent and cost-effective alternative for capturing genome-wide variation, particularly thanks to its high-throughput single nucleotide polymorphism (SNP) genotyping capabilities, enabling the rapid and extensive assessment of genetic diversity, population structure, and genetic relationships. In *B. oleracea*, GBS has been applied for multiple purposes, including the construction of high-resolution genetic maps, exploration of diversity in germplasm collections, and genotyping of near-isogenic lines (NILs) differing for specific traits. In cabbage, several GBS studies have focused on mapping valuable QTLs and fingerprinting commercial varieties [5,18,19]. Several investigations have exploited GBS-derived SNPs to explore diverse traits in *B. oleracea*, for instance heat tolerance in a segregating broccoli DH population [20]. Additionally, GBS was applied to broccoli NILs differing in leaf glossiness [21], and to identify QTLs associated with curding traits in Indian cauliflower germplasm [22]. A genetic diversity study of *B. oleracea* genotypes including broccoli, cauliflower, and Chinese kale used GBS to generate 21,680 high-quality SNPs from a panel of 85 landraces and improved genetic lines [7]. That study analyzed the population structure, phylogeny, and domestication patterns within landrace and cultivated broccoli, providing key insights into its evolutionary trajectory. A more recent study, based on SNP markers, used a wide germplasm panel including 912 samples of cultivated (landraces and cultivars) *B. oleracea*, wild *B. oleracea*, and C9-related *Brassica* species, providing evidence for two domesticated lineages, the “leafy head” lineage (LHL) and the “arrested inflorescence” lineage (AIL) [23].

Landraces are genetic materials that have been empirically selected by farmers over generations, well adapted to the specific environmental conditions in which they have been cultivated for a long time. In southern Italy, especially in the Puglia region, local varieties are still quite widespread and diversified [24,25]. In this region, high levels of variation have been documented in *B. oleracea* landraces [26], with some showing substantial differences from commercial cultivars. This study used GBS-derived SNP markers to evaluate the population structure and genetic relationships between these local varieties and known genetic material. The aim was to characterize distinctive landraces, accurately classify them within the correct taxonomic groups, and deepen our understanding of the overall relationships and evolutionary dynamics within this complex species.

## 2. Materials and Methods

### 2.1. Plant Material

**Figure 1:**
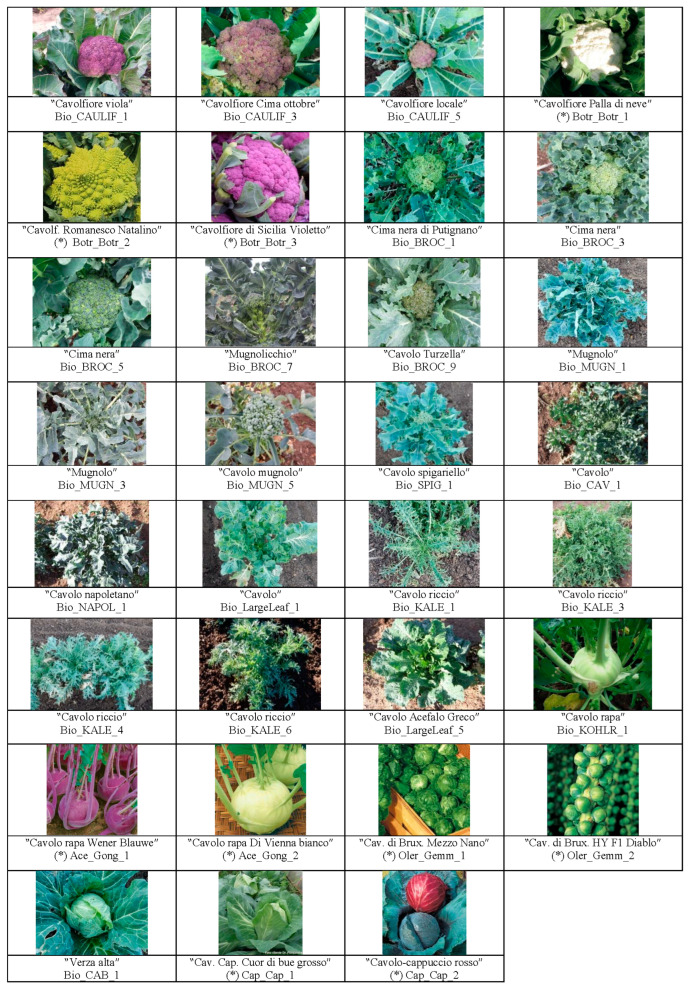
Selection of the *Brassica oleracea* varieties included in this study. Local or commercial names are shown, followed by codes (see Table S1), where the prefix Bio indicates landraces collected by the CNR-IBBR. Commercial cultivars (*) and their pictures were provided by the company Fratelli Ingegnoli SpA (https://www.ingegnoli.it/).

This study analyzed a collection of 82 *B. oleracea* genotypes (Table S1, Figure 1) representing diverse morphotypes, including broccoli (var. *italica*), cauliflower (var. *botrytis*), cabbage (var. *capitata*), Savoy cabbage (var. *sabauda*), Brussels sprouts (var. *gemmifera*), kohlrabi (var. *gongyloides*), Chinese kale (var. *alboglabra*), curly/ornamental kale (var. *sabellica*), other curly kale (var. *acephala*), and collard greens (var. *viridis*). Several *B. oleracea* landraces, some of which with uncertain subspecific classifications, were directly collected by scientists from the Institute of Biosciences and Bioresources (CNR-IBBR, Bari, Italy), specifically in the Puglia region, southern Italy. Due to a lack of morphological uniformity, two genotypes were included for some landraces to ensure a comprehensive representation.

### 2.2. GBS Assay and SNP Filtering

Genomic DNA was isolated from leaves and quantified as described in Sonnante et al. (2023) [27]. The DNA samples were then sent to the Genomic Diversity Facility at Cornell University (Ithaca, NY, USA) for library preparation, using the ApeKI enzyme. Sequencing was performed on an Illumina HiSeq2000 platform (100 bp reads) in a single lane, which included an empty, negative control sample. Reads were inspected to ensure barcodes matched 100% with expected sequences, then organized, de-multiplexed, and trimmed to first 64 bases from the enzyme cut site. The Discovery TASSEL-GBS pipeline [28], in combination with the *B. oleracea* genome version 1 (Plant GARDEN database) [10], was used to generate a variant call format (VCF) file. VCFtools (version 0.1.17) [29] was used to filter variants under specific criteria: variants with a minor allele frequency (MAF) below 0.05, mean depth of coverage under 5, or more than 80% missing data were removed. Additionally, indels were filtered out from the dataset. The SNP density plot was drawn using SRplot [30]. All GBS short reads were deposited in the NCBI Sequence Read Archive (SRA), public SRA accession number: PRJNA1138954.

### 2.3. Genetic Structure, Relationships, and F_ST_ Analyses

ADMIXTURE version 1.23 [31] was employed to examine population structure and assign individuals to genetic groups. The analysis included 10-fold cross-validation (CV) across potential sub-populations (K values) from 1 to 15 and, utilized 1000 bootstrap replicates to ensure robustness. Cross-validation scores were used to determine the optimal K value. Prior to analysis, SNP pruning was performed in PLINK with the following parameters: “--indep-pairwise 50 5 0.5”. Principal component analysis (PCA) was computed using the “--pca” option in PLINK (v1.90b6.24 64-bit) [32].

Phylogenetic relationships were assessed using the Neighbor-Joining method with a p-distance model in MEGA package v. 7 [33] with 1000 bootstrap replicates to ensure tree reliability. The resulting phylogenetic tree was imported and annotated with iTOL v. 6.9.1 [34].

The pairwise fixation index F*_ST_* on a per-site basis was estimated using the Weir and Cockerham formula [35], employing pairwise comparisons between two sub-populations. This estimation was performed using the VCFtools option “--weir-fst-pop”. SNPs with F*_ST_* values greater than 0.5 were maintained and mapped on the corresponding genes. To retrieve functional information on these genes, we performed a BLAST search against the *B. oleracea* proteome deposited in Ensembl Plants release 59, with gene features retrieved using *biomaRt* [36]. SNP impacts on gene sequences were assessed using SnpEff [37], applying default settings. Enrichment analyses for annotated gene ontologies [38] within the “biological process” domain were performed with *gprofiler2* [39], using a threshold = 0.05 and Benjamini-Hochberg FDR (False Discovery Rate) for multiple testing correction.

## 3. Results

### 3.1. Genotyping-by-Sequencing

Sequencing of the GBS library yielded approximately 264 million high-quality barcoded reads. Unique sequences, occurring at least three times after barcode processing, amounted to about 2.5 million. The initial raw VCF file contained 439,612 variants. After filtering operations, the number of SNP markers was reduced to 68,259. The missingness on a per-individual basis ranged between 19% and 42%. However, this parameter decreased significantly following the filtering procedures, ultimately never exceeding 6%. The mean depth per individual was notably high, with only eight samples having a depth below 100, while the remaining ones fell within the range of 100 to 996.

SNPs were categorized based on nucleotide substitution types: transitions (C↔T or A↔G) and transversions (A↔C, C↔G, A↔T, G↔T). Transitions were more frequent, comprising approximately 55% of the SNPs, while transversions accounted for 45%. The SNP density plot depicting the number of filtered SNPs in 0.1 Mb windows across the 9 chromosomes of *B. oleracea* is reported in Figure S1.

### 3.2. Population Structure

Population structure analysis was performed using a SNP-pruned dataset comprising 34,211 markers, with ancestral populations (K) ranging from 1 to 15. The cross-validation test indicated that K = 2 was the optimal value, representing the most accurate number of sub-populations based on individual ancestry estimates (Figure S2). With a high membership coefficient (q_i_ ≥ 80%), the results indicated that the majority of *B. oleracea* genotypes were classified as admixed, even though, at lower q_i_ values (≥60%), a distinct separation emerged between two categories: *B. oleracea* types used for their leaves, referred to as LHL, and those used for their flower heads, termed AIL (group 1 or 2, respectively, as illustrated in Figure S3). However, a group of landraces (including Bio_CAV), which could be considered belonging to the AIL lineage based on their morphological features, was admixed at K = 2.

**Figure 2:**
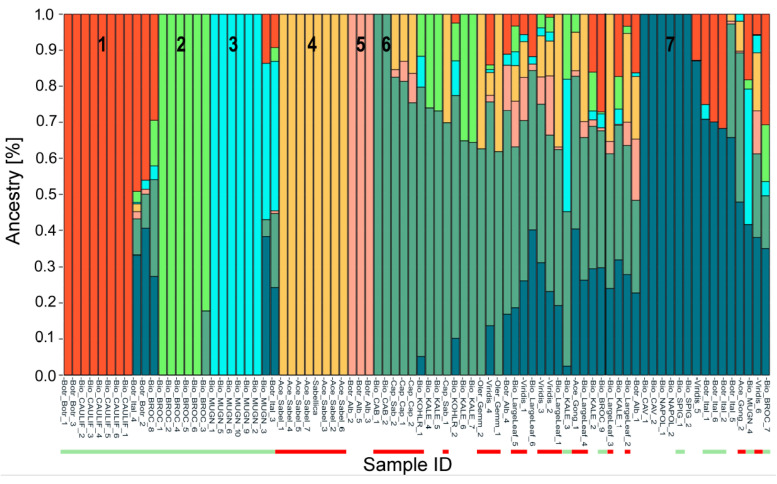
K7 model of population structure analysis based on 82 *Brassica oleracea* genotypes and 34,211 SNPs. The *y*-axis represents the estimated ancestry or membership coefficient (qi) for each genotype. Accession names are shown along the bottom of the figure (see Table S1). The different colors of the vertical bars correspond to the distinct groups (numbered from 1 to 7) identified at the K = 7 value. The green and red color bars beneath the sample names indicate the two groups, AIL and LHL, respectively, as observed at the K = 2 value (see Figure S3).

Nevertheless, to gain a clearer understanding of the morphotype groups, considering the genetic background of the germplasm under investigation (Table S1, Figure 1), we deemed K = 7 to be the best number of ancestry components for describing the relationships among the genetic material analyzed (Figure 2), although we used 10 morphotypes in this study. The population structure at K = 10, in fact, did not form 10 separate groups for the 10 morphotypes, but returned some anomalous clusters instead, e.g., kales with Savoy cabbage. At K = 7, with a q_i_ value ≥ 80%, group 1 (red) included the cauliflower landraces and cultivars, except for the Cavolfiore Romanesco Natalino (Botr_Botr_2), which was classified as admixed. Group 2 (lime) comprised the broccoli genotypes belonging to the Cima nera landrace (Bio_BROC_1–6). The third sub-population (light blue) was composed of the distinctive Mugnoli broccoli landrace (Bio_MUGN), characterized by a loose inflorescence compared to commercial broccoli (Figure 1). Cluster 4 (mustard) encompassed the kale genotypes classified as *B. oleracea* var. *sabellica*. Next, three Chinese kale genotypes were classified into group 5 (pink), while cabbage (var. *capitata*) and Savoy (verza) cabbage (var. *sabauda*) were included in cluster 6 (dark green). Group 7 (dark blue) encompassed the landraces Cavolo (Bio_CAV), Cavolo napoletano (Bio_NAPOL), and Cavolo spigariello (Bio_SPIG). Between clusters 6 and 7, 27 genotypes were identified as admixed.

### 3.3. Genetic Relationships Among Accessions

**Figure 3:**
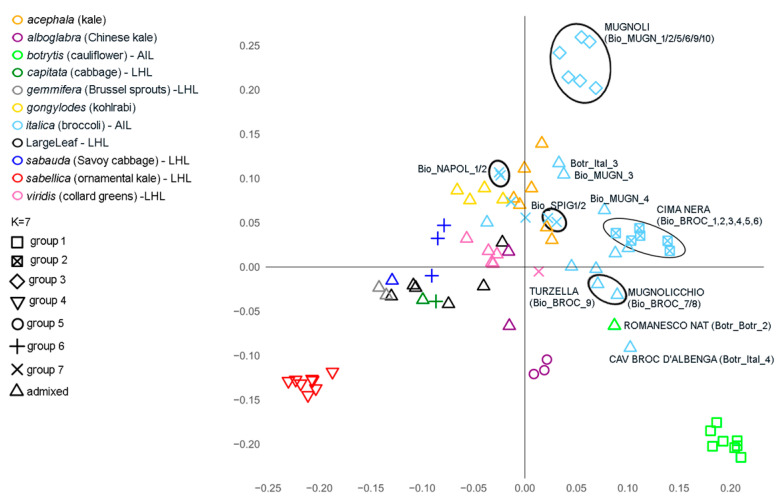
Principal component analysis (PCA). The plot illustrates the first two axes from a PCA conducted on 82 *Brassica oleracea* genotypes and 34,211 SNPs. The different colors correspond to the different *B. oleracea* morphotypes. “LargeLeaf” refers to samples arbitrarily named Bio_LargeLeaf, the classification of which was uncertain. AIL and LHL refer to “arrested inflorescence” and “leafy head” lineages, respectively; *acephala*, *alboglabra*, and *gongyloides* were not assigned to any lineages. The different shapes correspond to the distinct groups identified at K = 7 value of population structure analysis, including admixed genotypes. Specific genotypes referenced in the text are labeled by their common names alongside their sample identifiers (see Table S1).

In order to assess genetic relationships among the *B. oleracea* samples, principal component analysis (PCA) and phylogenetic analysis were performed. Upon initial examination of the PCA plot, it can be observed that the AILs are placed on the right side, while the LHLs are positioned on the left side of the graph (Figure 3 and Figure S4). Going into detail, the PCA reveals the formation of several distinct clusters within the *B. oleracea* germplasm under study. In the lower right part of the graph, the cauliflower group is clearly identifiable, consisting of six local varieties and two commercial cultivars. Moving upward to the central right side, the var. *italica* material is recognized, including the broccoli local varieties Cima nera and Mugnolocchio, and a commercial broccoli variety. Positioned between the cauliflower and the broccoli groups are the Romanesco Natalino cauliflower (Botr_Botr_2) and the Cavolo Broccolo d’Albenga (Botr_Ital_4).

In the upper right side of the chart, a distinct group of six var. *italica* genotypes is clearly defined. This group includes six Bio_MUGN samples from the Mugnoli landrace, which are noticeably separated from the other broccoli landraces (Bio_BROC). However, two genotypes classified as Mugnoli (Bio_MUGN_3 and 4), along with a Cavolo poverello (Botr_Ital_3) accession, are positioned between the upper Bio_MUGN group and the other broccoli genotypes. Two genotypes, locally known as Cavolo spigariello (Bio_SPIG), are located closer to the Bio_KALE genotypes (var. *acephala*). In the upper left part of the PCA plot, the four var. *gongylodes* genotypes (Ace_Gong and Bio_KOHL) are clustered together. In the central left section of the plot, the cabbage genotypes (Bio_CAB and Cap_Cap) are found.

The central lower portion of the plot displays a cluster of Chinese kales (var. *albogabra*). A distinct, compact group is on the lower left side of the plot, consisting of genotypes from var. *sabellica*. Moving upward, the plot reveals Oler_Gemm_1 and Oler_Gemm_2, two Brussels sprouts (var. *gemmifera*) cultivars, along with the LargeLeaf landraces, which are close to var. *viridis* samples.

**Figure 4:**
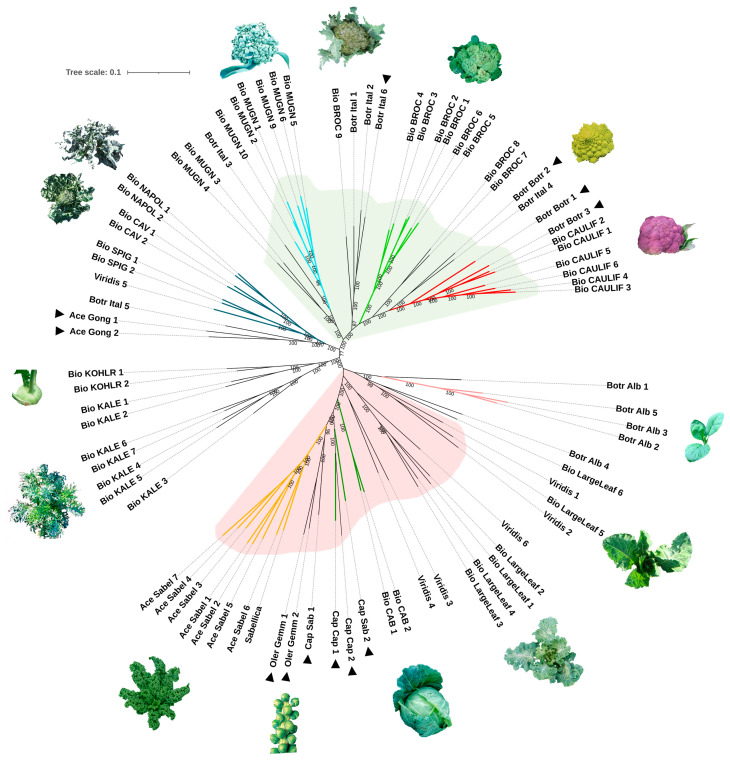
Neighbor-Joining phylogenetic tree of *Brassica oleracea* landraces. Numbers next to the branch nodes represent bootstrap values based on 1000 replications. The scale bar indicates an evolutionary distance of 0.1%. The light green and red backgrounds highlight the two main lineages supported by the population structure analysis at K = 2: the AIL and the LHL lineage, respectively. Colored branches refer to the distinct groups identified at K = 7 value of the structure analysis; black branches are admixed samples. Sample images correspond to the landraces or cultivars (black triangles) included in the major branches of the tree.

The Neighbor-Joining (NJ) clustering analysis produced a radial tree (Figure 4), starting in the upper right with small branches representing the entire set of cauliflower varieties and extending to the var. *sabellica* genotypes, on the lower left side. Apart from an intermediate section, the tree was predominantly divided into two main parts: the upper segment mainly containing the AIL types, and the lower section, predominantly made up of the LHL types. The first cluster includes eight cauliflower genotypes, followed by a small group consisting of Cavolo Broccolo d’Albenga (Botr_Ital_4) and Cavolfiore Romanesco Natalino (Botr_Botr_2). Next, the broccoli varieties are dispersed across several branches of the tree. The first broccoli group includes two genotypes belonging to the locally called Mugnolicchio landrace (Bio_BROC_7–8), while another cluster contains genotypes of the Cima nera (Bio_BROC_1–6) landrace. Further along, a branch features the broccoli landrace locally called Cavolo turzella (Bio_BROC_9), along with other landraces and a commercial hybrid. The subsequent clade includes multiple genotypes of the Mugnoli broccoli (Bio_MUGN), along with an Italian landrace obtained from the Leibniz Institute of Plant Genetics and Crop Plant Research (IPK, Gatersleben, Germany), named Cavolo broccolo poverello (Botr_Ital_3), which is another local name used for Mugnoli [40].

Following this section, the radial tree reveals a branch that splits into two sub-branches, predominantly containing genotypes from the LHL Cavolo riccio landrace (Bio_KALE_1–7). This grouping also includes various kohlrabi varieties (Bio_KOHL; Ace_Gong). The first cluster in the lower subdivision of the tree features a subgroup of Chinese kale (var. *albogabra*, Botr_Alb), alongside another subgroup consisting of var. *viridis*. Nestled among var. *viridis* samples are the LargeLeaf genotypes. Further along, the tree displays various cabbage types, followed by Brussels sprouts, and concludes with all genotypes belonging to var. *sabellica*, the curly/ornamental kale.

### 3.4. Molecular Signatures of Divergence

A total of 68,259 SNPs were analyzed to identify molecular signatures of divergence. In particular, we focused on divergent loci between the two sub-populations of *B. oleracea* genotypes: the leafy type LHL represented by the group 1 (red) in Figure S3, and the flowering head type AIL (group 2, green). To this purpose, we calculated the F*_ST_* for individual SNPs, setting the significance threshold at >0.50, as illustrated in the Manhattan plot (Figure S5). This analysis revealed 456 outlier SNPs, distributed across all 9 chromosomes of the *B. oleracea* genome. Using the available annotation for the *B. oleracea* genome, we found that 195 of these outlier SNPs were located within a total of 107 genes, 96 of which had 99 unique orthologs in *Arabidopsis* (Table S2). Overall, the processes associated with these genes included signal transduction and cellular communication; cell structure and mobility; cell division and organization; stress response and defense mechanisms; regulation of gene expression and RNA processing; as well as development and differentiation (Table S2). A functional enrichment analysis performed on these genes identified two significantly (*p* < 0.05) over-represented biological processes, both related to cell cycle phase transition. Among the genes with available orthology information in *Arabidopsis*, four exhibited the largest number of SNPs (N = 5 or N = 6) within each gene. In particular: (i) *Bo3g034090*, the ortholog of *AT2G28290* (*SYD*) in *Arabidopsis*, crucial for the development of the inflorescence meristem [41]; (ii) *Bo5g070760*, an ortholog of *AT1G43850*, encoding the SEUSS transcriptional co-regulator, which plays a critical role in floral development [42]; (iii) *Bo5g085170*, corresponding to *AT1G35460*, a BHLH1 transcription factor involved in the activation of other transcription factors implicated in flowering [43]; and (iv) *Bo9g113780*, an ortholog of *AT5G53890*, which encodes a leucine-rich repeat receptor kinase (LRR-RK) involved in the perception of phytosulfokine (PSK), a molecule that primarily promotes cellular proliferation [44]. A total of 40 missense SNPs, impacting 35 genes, were identified through comparison with the reference genome of *B. oleracea*. Among these genes, several key regulators were highlighted: *Bo8g020390*, orthologous to *AT1G46480*, which encodes WUSCHEL related homeobox 4, fundamental for stem cell maintenance, organ development, and overall plant growth [45]. The gene *Bo5g071270* is the ortholog of *AT1G47670*, encoding a transmembrane amino acid transporter family protein active in *Arabidopsis* flowers [46]. Additionally, we identified other genes with potential roles in flower development and cellular proliferation. For instance, *Bo2g150290*, the ortholog of *AT5G48880*, encodes a peroxisomal 3-keto-acyl-CoA thiolase 2 (KAT2), known for its important functions in inflorescence development [47], and *Bo5g071200*, which is orthologous to *AT1G47210*, encoding a cyclin-dependent protein kinase 3;2, and *AT1G47220*, encoding a cyclin A3;3 (Cyclin A3;3). Both are important for cell cycle regulation and contribute to flower development [48].

## 4. Discussion

Plant genetic resources for food and agriculture are crucial for preserving variation within crops, which supports resilience to environmental changes and productivity [49]. The *B. oleracea* species complex includes a wide array of crops that have been selectively developed for distinct traits through domestication and artificial selection processes. These traits include large arrested inflorescences for broccoli and cauliflower, leafy heads for cabbage, succulent stems for Chinese kale, and tuberous stem for kohlrabi. Over time, farmers have developed a myriad of landraces for each crop type, with Italy recognized as a center of genetic diversity for several *Brassica* crops, including var. *botrytis* (cauliflower) and var. *italica* (broccoli) [50].

In this study, we used GBS-derived SNPs to analyze landraces from the Puglia region, Italy, aiming at understanding their relationships with other local and commercial varieties. Additionally, by including a broad range of *B. oleracea* morphotypes in our dataset (Table S1), we sought to investigate patterns of relationships and evolutionary dynamics within this species complex. By focusing on SNPs located in genes potentially implicated in the divergence between LHL and AIL varieties, we aimed at elucidating the molecular signatures that underpin these distinctions.

The distribution of morphotypes in our phylogenetic tree, coupled with the results obtained from the structure and PCA analyses, largely aligns with findings from recent comprehensive studies based on sequence polymorphisms across the genomes of different morphotypes [17,23]. In those investigations, three primary groups were identified: the “arrested inflorescence” lineage (including broccoli and cauliflower) on one side, the “leafy head” lineage (such as cabbage, collard greens, and ornamental kales) on the other, and kales situated between these two groups.

In our study, a closer examination of individual varieties revealed that several landraces from Puglia form distinct clusters in the phylogenetic tree. For instance, most of the Mugnoli broccoli genotypes form a distinct group that is well separated from both other landraces and from commercial broccoli varieties. Compared to standard broccoli, Mugnoli are characterized by a looser arrested inflorescence featuring larger (white or yellow) flowers (see Figure 1). The Mugnoli broccolis, first described in a book on horticulture in the Salento area (southern Puglia, Italy) [51] and detailed in a later study [39], are hypothesized to be either an ancestral form of broccoli or a result of parallel evolution. In our phylogenetic tree, the Mugnoli genotypes occupy a basal position relative to the broccoli–cauliflower cluster, suggesting that they might represent a more primitive form of domestication. This landrace has been cultivated for a long time in Salento, an area renowned for preserving a rich diversity of vegetable landraces [24,52,53].

Another notable group of broccoli includes six genotypes from the Cima nera landrace (Bio_BROC 1–6). Grown in the central part of the Puglia region (southern Bari province), this local variety is more similar to commercial broccoli than Mugnoli. However, it possesses peculiar features, first described by Bianco and Pimpini (1990) [54], that set it apart from other broccoli varieties, highlighting its unique attributes (see Figure 1; [26]). On the other hand, the Turzella landrace (Bio_BROC_9) groups with other broccoli landraces and a hybrid cultivar in our analysis, whereas Mugnolicchio (Bio_BROC_7–8), which is cultivated in a restricted area (Altamura) and is at risk of extinction [26], forms a distinct cluster that lies between the other broccolis and the cauliflowers. This landrace is characterized by smaller, less compact flower heads, and larger white flowers compared to standard broccoli cultivars, as well as by sweet and aromatic organoleptic qualities [50].

Interestingly, the Cavolo broccolo d’Albenga (Botr_Ital_4) and the cauliflower Romanesco Natalino (Botr_Botr_2), identified as admixed in the structure analysis, in both the PCA and the phylogenetic tree are nestled between the broccoli and the cauliflower groups. Cavolo broccolo d’Albenga is an IPK accession of Italian origin, classified as var. *italica*, while the Romanesco Natalino is a well-known commercial cauliflower variety. Although we did not grow Cavolo broccolo d’Albenga ourselves, online images reveal that its flower head resembles the Romanesco Natalino cauliflower, characterized by a conic shape of curds and a peculiar fractal geometry (see Figure 1). This resemblance may be attributed to an accelerated rate of meristem production, which can lead to an increased number of internodes in the meristem axis [55]. The observation that one variety is labeled as broccoli and the other as cauliflower, coupled with their position in both the PCA and the phylogenetic tree between var. *italica* and var. *botrytis*, suggests that this *B. oleracea* type with conic curds may exhibit intermediate traits between these two crops. Indeed, a previous study examining landraces and improved varieties of broccoli and cauliflower found that a Romanesco cauliflower was phylogenetically grouped with a cluster of broccoli landraces (see Figure 1 in [7]). Moreover, Cai and colleagues (2022) [23] found that several broccoli clades were interspersed with Romanesco cauliflowers. All these pieces of evidence might indicate a potential blending of traits between broccoli and cauliflower in the Romanesco variety. 

The landraces known as Cavolo reale, Cavolo acefalo greco, or simply Cavolo, arbitrarily designated as Bio_LargeLeaf, were found to be interspersed among var. *viridis* accessions, indicating their potential classification within this taxonomic group.

In the population structure analysis, the cross-validation test identified K = 2 as the most accurate representation of sub-populations based on individual ancestry estimates. Therefore, we focused on SNPs with high F*_ST_* values distinguishing the two main groups, LHL and AIL. This analysis led to the identification of several interesting genes, many of which have homologs in *Arabidopsis* known to play crucial roles in relevant processes such as cell differentiation, flowering, etc. For instance, the ortholog of *Bo3g034090*, *AT2G28290*, encodes a SWI2/SNF2-like protein belonging to the SNF2 subclass, one of three classes of SWI/SNF ATPases in plants, and serves as a core enzyme in SYD-associated SWI/SNF (SAS) complexes [56]. Lines of evidence suggest that during flower development, the SWI2/SNF2 ATPases are recruited to the regulatory regions of AP3 and AG, physically interacting with two key transcriptional activators, LEAFY (LFY) and SEPALLATA3 (SEP3) [57]. Another notable example is the ortholog of *Bo5g070760*, *AT1G43850*, which encodes SEUSS (SEU), a transcriptional co-regulator of AGAMOUS. SEU functions alongside LEUNIG to repress AG expression in the outer floral whorls. Functional analyses have demonstrated SEUSS critical roles in floral organ identity specification, floral meristem termination, and gynoecial development [58]. Ectopic expression of AG in the perianth has been shown to cause homeotic transformations of perianth organs, as well as a variety of additional pleiotropic phenotypes observed in *seu* mutants [42]. Subsequently, the same research group provided evidence of the involvement of SEUSS and LEUNIG in regulating cell proliferation, vascular development, and organ polarity in *Arabidopsis* petals. They also demonstrated that these factors influence petal size and shape via AGAMOUS-independent mechanisms [59]. SEU is also involved in specifying floral organ identity by repressing AG, as well as promoting ovule formation in a specialized meristematic tissue known as the carpel margin meristem (CMM) [60].

Furthermore, we identified the gene *Bo2g150290*, an ortholog of *AT5G48880*, which encodes a peroxisomal 3-keto-acyl-CoA thiolase 2 (KAT2). This enzyme plays a critical role in all known aspects of peroxisomal β-oxidation, which is essential for inflorescence development in *Arabidopsis* [47]. The plant hormone jasmonic acid (JA), which plays a key role in flower development, is synthesized and matured through β-oxidation. In *B. oleracea*, the exogenous application of JA and its methyl ester has been shown to stimulate growth and enhance the nutritional potential of edible heads across various morphotypes [61]. Additionally, we identified a gene encoding a leucine-rich repeat receptor kinase (LRR-RK), which, in *Arabidopsis*, is involved in the perception of phytosulfokine (PSK), a signaling molecule that primarily promotes cellular proliferation. Triple mutations in the *AtPSK* gene resulted in dwarfism due to reductions in both cell number and cell size [44]. In *Arabidopsis*, seven precursor genes (*AtPSK1-AtPSK7*) encoding PSK have been identified. Among them, *AtPSK4*, is expressed in various tissues, including leaves and flowers; *AtPSK5* is mainly expressed in flowers; and *AtPSK7* is specifically expressed in siliques and seeds [62].

## 5. Conclusions

In conclusion, this study allowed us to highlight the morphological and molecular diversity still preserved in a specific region of southern Italy by analyzing *B. oleracea* landraces and different morphotypes. Moreover, this research has provided useful tools to accurately assign previously unidentified material to the appropriate taxonomic classifications. Additionally, while further studies are needed to explore the functions of the genes harboring SNP markers with the highest F*_ST_* values, this work offers valuable insights into the potential molecular modifications that may contribute to the development of a particularly complex inflorescence in *B. oleracea*.

## Data Availability

GBS short reads were deposited in the NCBI Sequence Read Archive (SRA) under BioProject PRJNA1138954.

## Supplementary Materials

The following supporting information can be downloaded at: https://www.mdpi.com/article/10.3390/plants14010020/s1, Figure S1: SNP density plot depicting the number of filtered SNPs in 0.1 Mb windows across the 9 chromosomes of *Brassica oleracea*; Figure S2: Cross-validation (CV) error estimates on each K tested on the 82 *Brassica oleracea* samples; Figure S3: K2 model of population structure analysis based on 82 *Brassica oleracea* genotypes and 34,211 SNPs. The *y*-axis represents the ancestry or estimated membership coefficient (q_i_) for each genotype. Accession names are shown along the bottom of the figure (see Table S1). The different colors of the vertical bars correspond to the distinct groups (numbered 1 and 2) at K = 2 value. The red and green color bars beneath the sample names indicate the two lineages, LHL and AIL, respectively, obtained when considering a q_i_ ≥ 60%.; Figure S4: PCA—principal component analysis (PCA). The plot illustrates the first two axes from a PCA conducted on 82 *Brassica oleracea* genotypes and 34,211 SNPs. The different colors correspond to the different *B. oleracea* morphotypes. “LargeLeaf” refers to samples arbitrarily named Bio_LargeLeaf, the classification of which was uncertain. The different shapes correspond to the distinct groups identified at K = 2 value of population structure analysis, including admixed genotypes. Specific genotypes referenced in the text are labeled by their common names alongside their sample identifiers (see Table S1). Figure S5: Manhattan plots depicting the results of F*st* outlier tests between sub-populations of *Brassica oleracea*, specifically leafy type (LHL), and flower head type (AIL). The *x*-axis represents the chromosomes of *B. oleracea*, while the *y*-axis displays the F*st* values for individual SNPs. Dashed red and green lines delineate the significance thresholds of 0.5 and 0.8, respectively; Table S1: *Brassica oleracea* plant material used in this study. The prefix Bio refers to landraces collected by CNR-IBBR. N.A.: Not available data; Table S2: SNPs with pairwise fixation index (Fst) value > 0.50. Gene ID, description, and annotated orthologs in *Arabidopsis*.
