# Supplementary material for: Genetic Relationships and Molecular Signatures of Divergence in Traditional Landraces and Morphotypes of Brassica oleracea
Source: Plants (Basel). 2024 Dec 25;14(1):20. doi: 10.3390/plants14010020 (PMC11722934; doi:10.3390/plants14010020)
Supplement: Supplementary file 1 [file plants-14-00020-s001.zip › Supp_Figures.pdf]

SUPPLEMENTARY FIGURES

Genetic Relationships and Molecular Signature of Divergence in Traditional Landraces and Morphotypes of *Brassica oleracea*

Diana L. Zuluaga [1], Nunzio D'Agostino [2], Emanuela Blanco [1], Pasquale L. Curci [1] and Gabriella Sonnante [1,*]

[1] Institute of Biosciences and Bioresources, National Research Council, Via Amendola 165/A, 70126 Bari, Italy; diana.zuluaga@ibbr.cnr.it (D.L.Z.); emanuela.blanco@ibbr.cnr.it (E.B.); pasqualeluca.curci@ibbr.cnr.it (P.L.C.)
[2] Department of Agricultural Sciences, University of Naples Federico II, Piazza Carlo di Borbone 1, 80055 Portici, Italy; nunzio.dagostino@unina.it

* Correspondence: gabriella.sonnante@ibbr.cnr.it

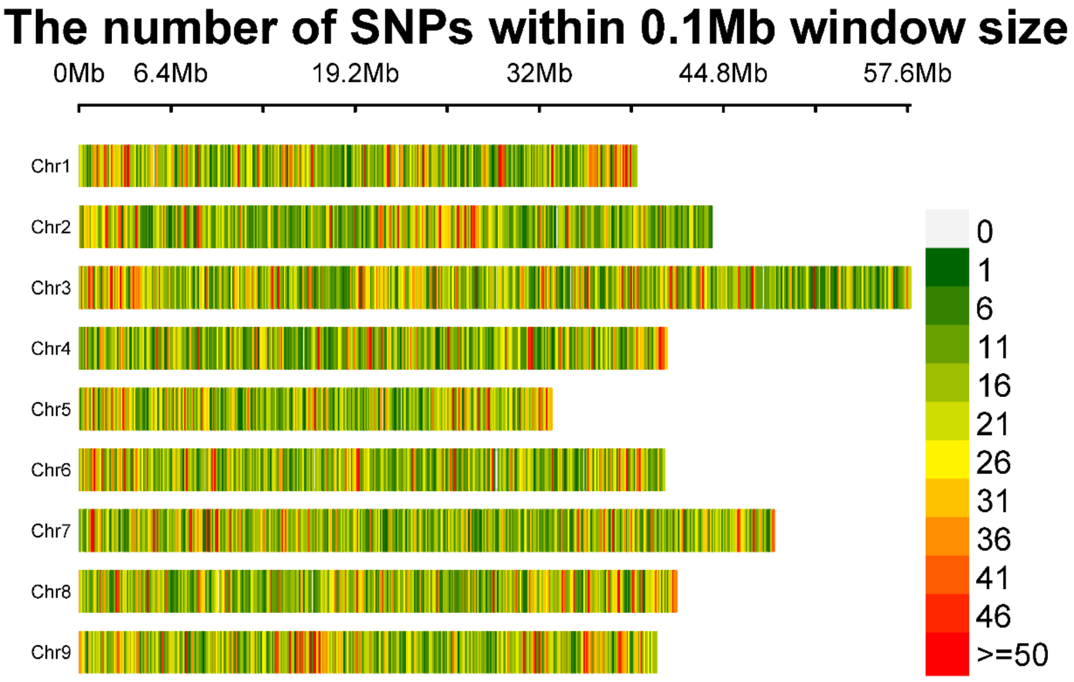

Figure S1. SNP density plot depicting the number of filtered SNPs in 0.1 Mb windows across the 9 chromosomes of *Brassica oleracea.*

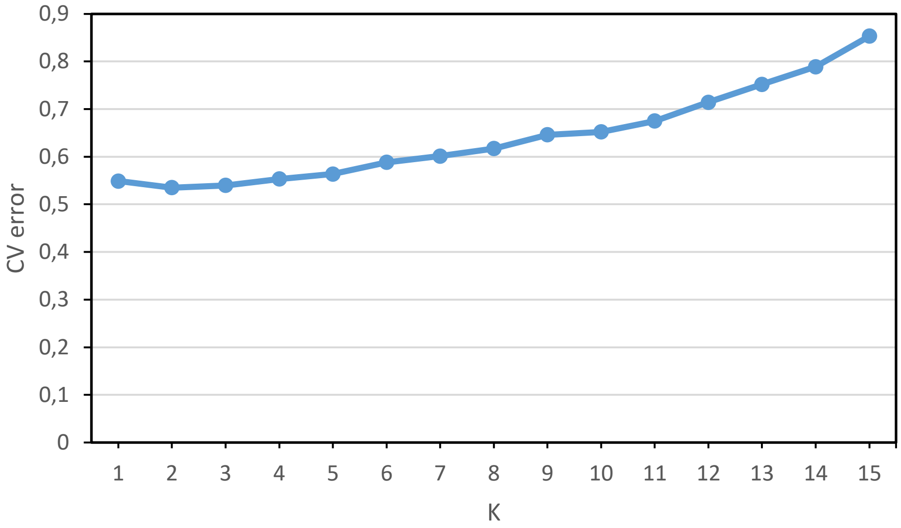

Figure S2. Cross-validation (CV) error estimates on each K tested on the 82 *Brassica oleracea* samples.

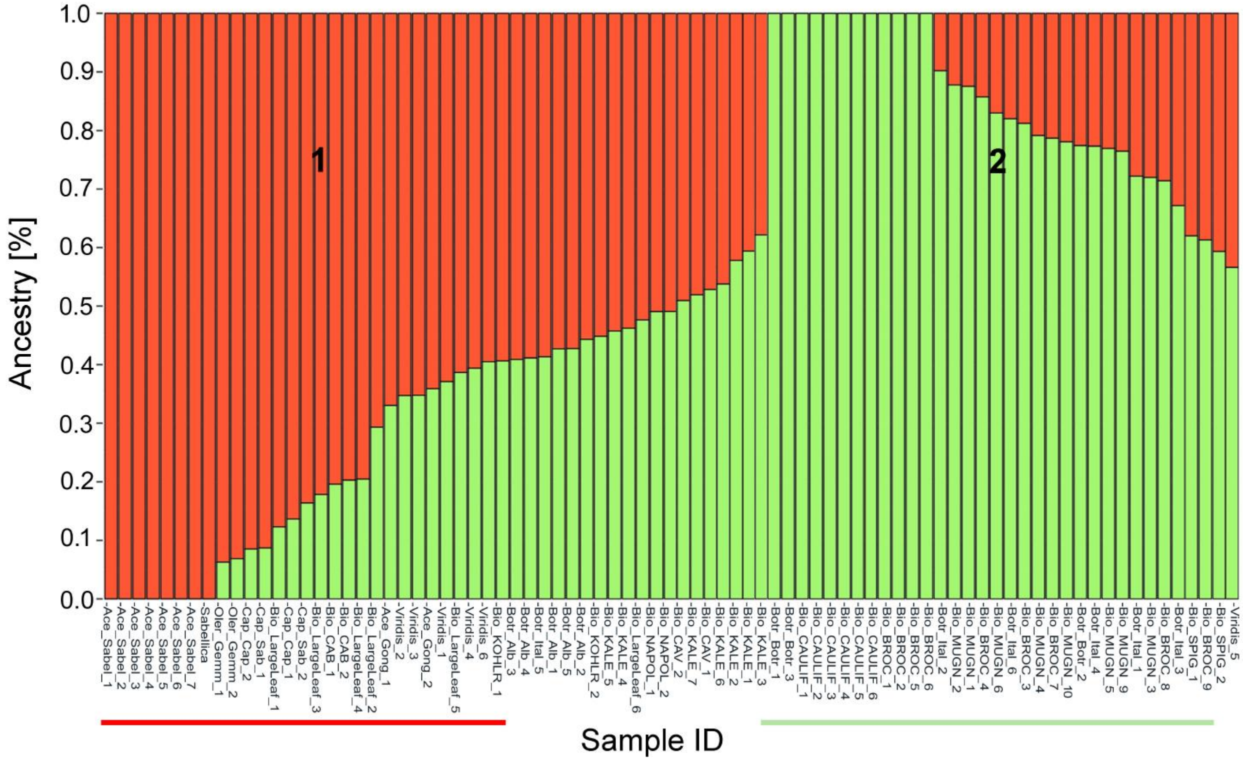

Figure S3. K2 model of population structure analysis based on 82 Brassica oleracea genotypes and 34,211 SNPs. The y-axis represents the ancestry or estimated membership coefficient (qi) for each genotype. Accession names are shown along the bottom of the figure (see Table S1). The different colors of the vertical bars correspond to the distinct groups (numbered 1 and 2) at K = 2 value. The red and green color bars beneath the sample names indicate the two lineages, LHL and AIL, respectively, obtained when considering a qi ≥ 60%.

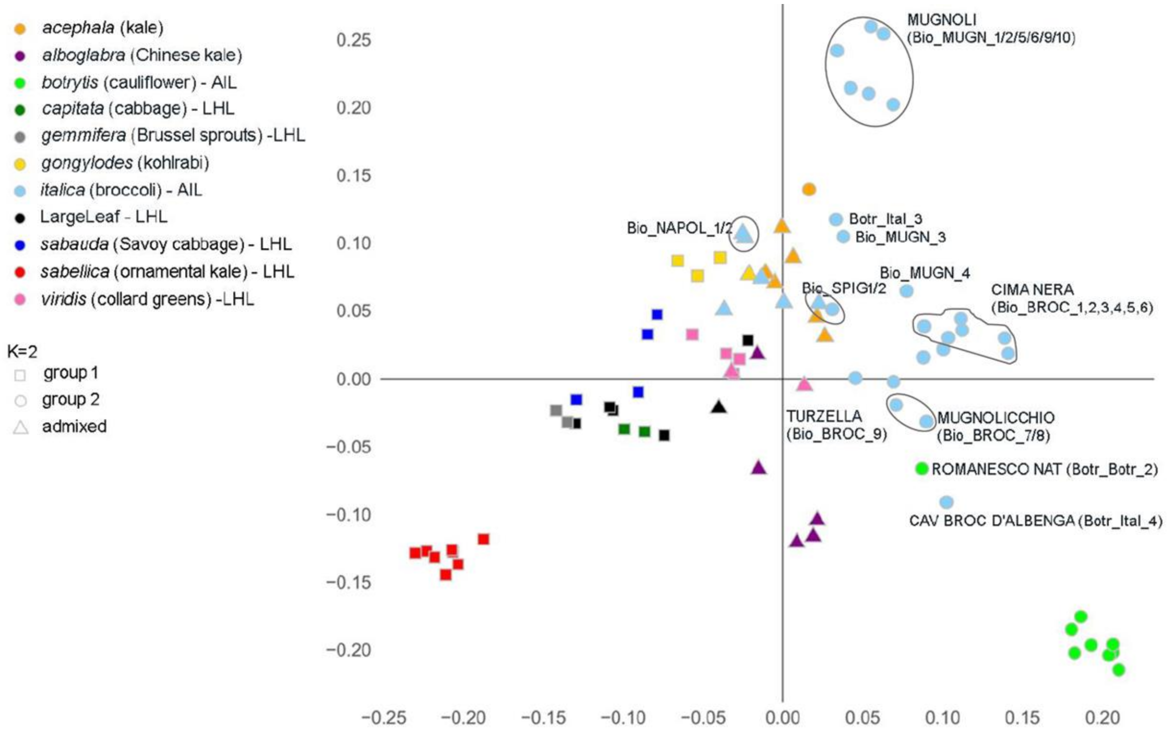

Figure S4. PCA Principal component analysis (PCA). The plot illustrates the first two axes from a PCA conducted on 82 *Brassica oleracea* genotypes and 34,211 SNPs. The different colors correspond to the different *B. oleracea* morphotypes. “LargeLeaf” refers to samples arbitrarily named Bio_LargeLeaf, the classification of which was uncertain. AIL and LHL refer to “arrested inflorescence” and “leafy head” lineages, respectively; acephala, alboglabra, and gongyloides were not assigned to any lineages. The different shapes correspond to the distinct groups identified at K=2 value of population structure analysis, including admixed genotypes. Specific genotypes referenced in the text are labeled by their common names alongside their sample identifiers (see Table S1).

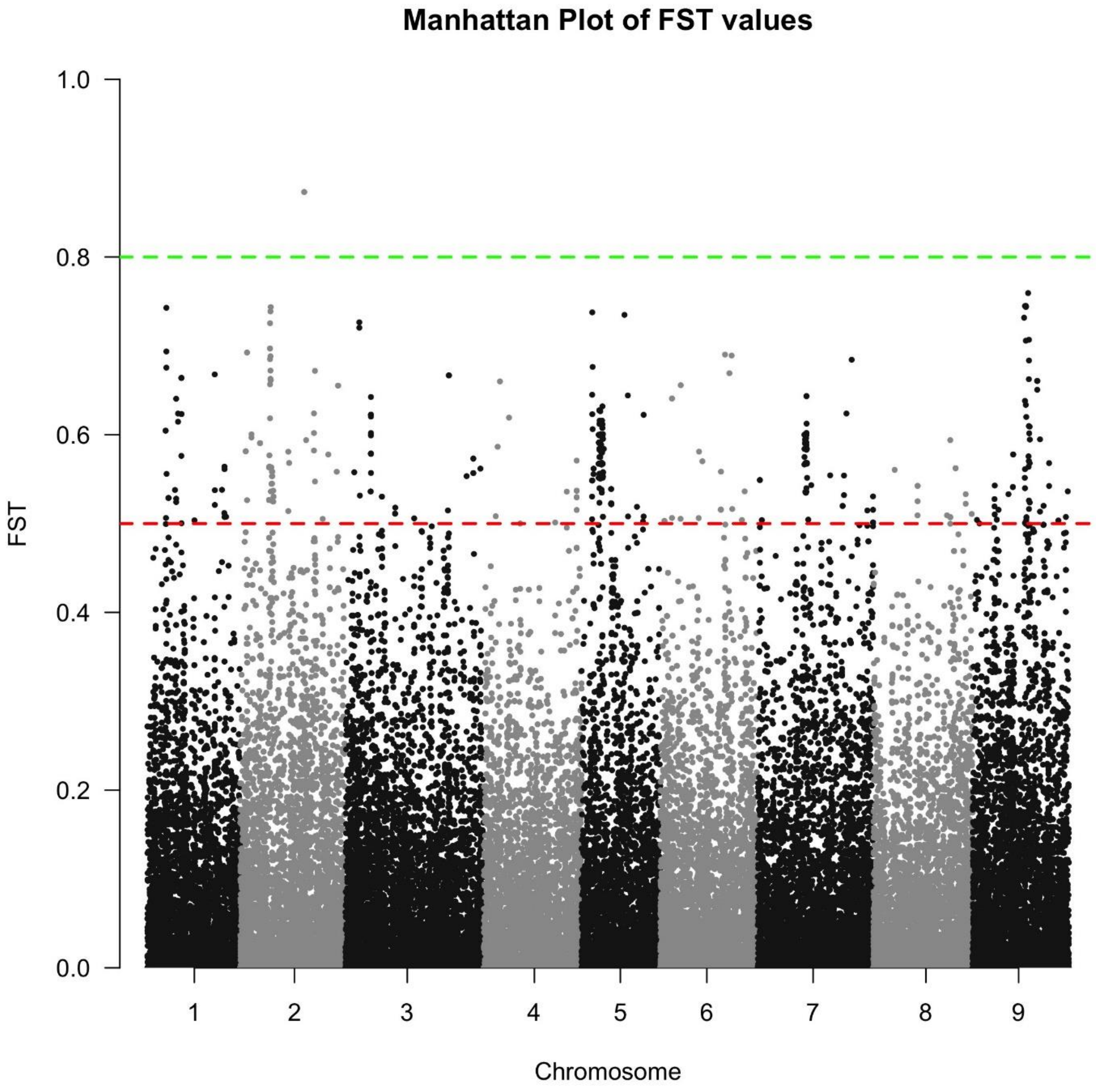

Figure S5. Manhattan plots depicting the results of F*st* outlier tests between sub-populations of *Brassica oleracea*, specifically LHL and AIL. The x-axis represents the chromosomes of *B. oleracea*, while the y-axis displays the F*st* values for individual SNPs. Dashed red and green lines delineate the significance thresholds of 0.5 and 0.8, respectively.
